# Board Gender Diversity, Corporate Social Responsibility Disclosure, and Firm’s Green Innovation Performance: Evidence From China

**DOI:** 10.3389/fpsyg.2022.892551

**Published:** 2022-06-24

**Authors:** Khwaja Naveed, Cosmina L. Voinea, Nadine Roijakkers

**Affiliations:** Faculty of Management, Open University of the Netherlands, Heerlen, Netherlands

**Keywords:** board gender diversity, corporate social responsibility, green innovation, social resilience theory, role congruence theory

## Abstract

The current research investigates the interplay of board gender diversity (BGD), the quality of corporate social responsibility disclosure (CSRD), and the green innovation performance (GIP) of a firm. It examines the moderation effect of the CSRD on the relationship between corporate GIP and BGD. The study inculcates 3,736 firm-year observations of A-share listed Chinese firms from 2010 to 2019. Least square dummy variables method, generalized method of moments, and 2SLS are employed for the analysis of the study. The findings foster an affirmative and significant impact of BGD on corporate GIP in terms of green innovation patents. Moreover, the quality of CSRD is also detected for a significant moderating effect on the relationship between BGD and corporate GIP. The quality of CSRD emerges to be an indicator for social resilience and female role congruence under the purview of the social resilience theory and the role congruence theory, respectively. This research would help managers and policymakers of developing nations in formulating environmental innovation strategies for corporate sustainability.

## Introduction

Sustainability literature has probed environmental concerns in different premises to unearth an optimal mix of sustainable activities in the throes of grappling with the recent climate change. Lopatta et al. (2020) reported innovation as a superior player in this triage as it not only mitigates the production of pollutants but also increases cost efficiency. The urge from stakeholders to operate in an environment-friendly manner has also pushed the firms to integrate green practices into the strategic and process management of firms.

Innovation and green practices converge in green innovations (GIs) which encompass all facets of innovation concerning green products and processes to manage the environment, energy usage, pollutant production, and waste disposal and recycling (Chen et al., 2006). Alongside, corporate social responsibility obliges firms to respond to the legitimacy urge from different stakeholders enticed by the socio-environmental flux.

The promulgation of GI is a strategic issue for firms, dependent upon different internal and external factors ranging from stakeholders’ pressure to consumer awareness (Nadeem et al., 2020) and the dynamics of the board of directors (Dixon-Fowler et al., 2017). As corporate governance (CG) concerns strategic issues, the conflicting interests of different actors in the environmental sustainability and GI are a vital challenge for firms that require appropriate design and triage of CG practices. The policies and procedures of a firm emanate from its board of directors (BODs); therefore, the structure, function, and composition of the BODs are the gist of CG. It is thus crucial to ascertain the drivers of GI in terms of the characteristics and dynamics of the BOD.

Board gender diversity (BGD) is an extensively researched construct in the extant literature on CG, which has gained substantial attention in academia, regulatory bodies, and the media (Hülsbeck et al., 2019). Laws concerning the mandatory quota of women on corporate boards have been pioneered in various countries to assure BGD. In the case of environmental sustainability and GI, the arena of stakeholders is vast, and therefore, the polyphonic minutiae of BGD in terms of its differentiated impact on different sustainability outcomes are acclaimed (Bradley and Klein, 2016). The growing fraction of Chinese firms with better BGD, increasing to approximately 70% of firms, fosters its paramount part in CG, which entails due deliberation as regards GI.

Focusing on environmental performance, empirical evidence on BGD has mixed findings whereby a majority of the studies report a positive and significant relationship between gender-based diversity and different environmental sustainability constructs (Post et al., 2015; Orazalin and Mahmood, 2021). However, some studies report no significant relationship between them (Nadeem et al., 2020; Nguyen et al., 2021), and a scant number of studies even suggest a negative relationship (Orazalin and Baydauletov, 2020).

Corporate social responsibility disclosure (CSRD) is a report disseminated by a firm to inform the relevant stakeholders regarding the policies and activities it adopts toward social and environmental responsibility in the purview of its economic activity and corporate performance (Gray et al., 1995; García-Sánchez et al., 2019). CSRD has a cost in terms of sundry resources; however, due to its financial and nonfinancial benefits, it has become a norm for responsible firms (Issa and Fang, 2019). The quality of these disclosures determines as to how they are perceived by the stakeholders because credibility and opportunistic behavior (use of CSRD as an impression management tool) have been reported as vital factors in CSR decoupling (Chen et al., 2016). High-quality CSRD is reported to be beneficial in trust enhancement of investors, which leverages firms with ease in obtaining capital, curbing constraints in financing, attenuating information asymmetry, and improving reputation (Dhaliwal et al., 2011; Cho and Patten, 2013). Pertaining to the trust of investors, Zahller et al. (2015) have put forward the theory of social resilience, which posits a shock-absorbing capacity of firms when they publish quality CSRD.

Given the constraining factors in the relationship between BGD and GI, quality CSRD has the potential to compensate for them in the purview of role congruence and social resilience theories while instilling an enabling context for the positive role of female directors in the GI.

This study will contribute to the body of knowledge in the following ways: First, most of the current studies focus on the linkage between BGD and environmental performance in different institutional contexts; however, the firm-level context is still in veil. We are attempting to fill this void in terms of CSRD. Second, this research contributes to the current literature by investigating the association between BGD, CSRD quality, and corporate green innovation performance (GIP) while considering the scenario of China, an intensively industrialized market. Our findings may provide new ways to impact corporate GIP through BGD and CSRD quality.

The following is a representation of the structure of the article. The theoretical framework and hypothesis formulation are presented in section “Theoretical Framework and Hypothesis Development.” The sample, variables, empirical models, and procedure are all described in section “Data, Measurement, and Research Methodology.” Section “Result and Discussion” discusses the empirical findings. The conclusion is presented in section “Conclusion.”

## Theoretical Framework and Hypothesis Development

The theoretical underpinning of studies involving BGD and sustainability has traditionally been revolving around the stakeholder, resource dependence, upper echelons, and legitimacy theories. CSRD, on the other hand, has majorly been considered under the stakeholder, legitimacy, and signaling theories (Bannò et al., 2021).

The factors reported in the extant literature for a significant effect on studies involving sustainability constructs and board diversity are contextual ones, which can be categorized into (a) external pressures, such as stakeholders’ pressure and regulations; (b) firm-level factors, such as firm age and firm size; and (c) internal factors, such as personality traits of board members.

The legitimacy theory complemented by stakeholder and signaling theories describes the environmental and social activities of firms as a response to the demands of the society and stakeholders (Chiu and Sharfman, 2011). Green innovation is also regarded as a legitimizing measure by the firms through which an environment caring signal is sent to the stakeholders to envisage a proactive image of the firm among the stakeholders (Frondel et al., 2008). Empirical studies also support this argument of garnering environmental legitimacy (Berrone et al., 2013). However, firms differ in terms of sustainability response while having homogeneous institutional settings and legitimacy pressures.

Corporate boards play a major role in catering to the critical resources of the firms in the form of advice, consultation, and legitimacy (Hillman and Dalziel, 2003). Gender diversity of corporate boards expands the scope of a firm in terms of networks and linkages to other firms (Hambrick, 2007). Also, Milliken and Martins (1996) have reported the dependence of appropriate decision-making on differentiated problem-solving styles as it leverages an improvement in communication, a wider range of perspectives, and a more detailed critical analysis of the issues. Glass and Cook (2016) suggested that heterogeneity in the form of diversity is more liable for environment caring policy innovation than unanimity in the form of homogeneity.

The mixed results of the studies involving BGD and sustainability however still demand a theoretical probe into their relationship. To address this issue, we have inculcated the role congruence and social resilience theories of Zahller et al. (2015).

Organizational resilience is the ability of a firm to foresee impending threats, to handle “out of the blue” events effectively, and to internalize the emergent dynamic capabilities acquired in the due process for long-term success and competitive advantage (Coutu, 2002; Sheffi and Rice, 2005; Duchek, 2014). Perceived organizational legitimacy typifies the perception of stakeholders regarding the values, aspirations, and culture of the firm pertaining to the use and exploitation of scarce resources, regulatory competence and compliance, ethical and fair conduct of employees, and conscientious transaction with clients, among others. (Freeman, 2007; Lindblom, 1994). As CSRD has been considered an effective tool of legitimization for firms (Muñoz-Torres et al., 2013), its quality will also lead to organizational resilience and catre to the shocks which shapes the differing and mixed results of the BGD–GI relationship.

In the same manner, the role congruence theory argues for an enabling environment pertaining to women’s gender-specific performance. It has been reported that, if the board culture is suppressive of the gender-specific traits of women, it will misalign them in the form of backlash avoidance behaviors (Vial et al., 2016), and they will prove counterproductive for the green innovation’s strategic upheaval (Triana et al., 2014).

### Board Gender Diversity and Corporate GIP

Green innovation as a confluence for environmental performance and productivity is proving a long-term competitive advantage for firms (Castellacci and Lie, 2017). The outset of this concept entails product and process innovation with relatively less environmental hazards while using natural resources (Bartlett and Trifilova, 2010).

The environmental regulations have been increased in the recent promulgation of global concerns regarding climate change, which has increased the liabilities of firms in the form of regulatory compliance (Bansal and Clelland, 2004). The liabilities entice pressures that are reported to be influencing the outline of competitive advantage while promoting environment-friendly investments on top of the agenda of boards in the purview of sustainability (Chang et al., 2011). Stakeholders’ pressure has also pressed the firms to operate in an environment-friendly manner and integrate green practices into the firms’ products and processes (Kawai et al., 2018). The corporate sector has responded to these pressures through different interventions whereby green innovations have received high appreciation in the triage for long-term competitive advantage and sustainability (Chu et al., 2018).

Production/service innovation imperatively requires the consideration of consumer preferences for its success; therefore, the “Going greener” reflection needs to be converged with the dominion of the consumers in the case of green product/service innovations. On the other hand, process innovation concerns modification in the complete production process (Reichstein and Salter, 2006), and therefore, the “Going greener” reflection needs to be embedded in the entire modification milieu. As a second-order innovation activity, process innovation seems to be an unattractive activity; however, it fosters a long-term competitive advantage for a firm due to its inimitable nature (Pisano and Shih, 2012). The “Going greener” reflection necessitates the transformation of management conviction, organizational design, and production modus operandi, which entails an open and collaborative working environment (Ramos et al., 2018).

Women are contended to raise the deliberation of environmental issues on corporate boards amid their benevolence and empathy (Post et al., 2011); therefore, BGD can be termed as an important CG construct in terms of green innovation (GI) activities (Xu et al., 2020). Also, the leadership style of women is a participative one, which potentially kindles increased information exchange in BOD discussions (Adams and Ferreira, 2009) and may upgrade the effectiveness of the strategic changes required for green innovations. It has been reported that women are more heady concering “Community Influentials” (directors with enhanced sustainability and ecological orientation) than the male members of BODs (Rehbein et al., 2013).

Moreover, the stakeholders’ engagement requires a communal acumen and relationship sensitivity, which are the traits being attributed to women in the extant literature on strategic management and corporate governance (Hillman et al., 2000; Robinson and Lipman-Blumen, 2003; Nielsen and Huse, 2010). In tandem with reasoning, Post and Byron (2015) have reported the positive association of BGD with the BOD’s enhanced strategic consideration of environmental and sustainability concerns. In an environment of uncertainty, the perceptive skills of women on board are also contended to be efficient in the triage among different stakeholders (Naveed et al., 2021). The efficiency of women on board in the trade-off between stakeholders’ value and interest has been reported in the empirical studies of Miller and del Carmen Triana (2009) and of Kim and Starks (2016). Keeping in view the aforementioned argumentation, we hypothesize our first hypothesis as follows:

Hypothesis (H1): BGD has a positive relationship with corporate GIP.

### CSRD and GIP

The antecedents of green innovation are clustered into three categories in the extant literature: (a) contextual factors, mainly referring to various external pressures, such as social norms, regulation, and stakeholder pressure; (b) firm-level characteristics, such as firm size and corporate financial performance; and (c) internal factors, which mainly refer to the individuals’ characteristics on a board.

CSRD is a powerful tool that informs relevant stakeholders about the social and environmental activities of firms (García-Sánchez et al., 2019) while premising the existence of the firm associated with the prerogative of the society, instead of an inherent right of the firm itself (Deegan, 2002). Also, the CSRD quality confirms the privilege of the political legitimacy to firms required for their successful business operations (Rauf et al., 2021b). Moreover, the CSRD quality ascertains the compliance of firms toward their social responsibility in terms of global standards (Chen et al., 2016) and is reported for significant association with the confidence of investors, information asymmetry, and betterment in repute (Cho and Patten, 2013).

Pertaining to green innovations, the quality of CSRD is contended to affect it through the amelioration of agency problems and through the formal and informal institutional isomorphism. When information asymmetry is alleviated in case of good-quality CSRDs, it increases the monitoring role of controllers in the purview of the principal–agent dilemma (Wang et al., 2016). Aghion et al. (2013) have described this incentive for innovation from the perspective of a career concern model. The Porter hypothesis regarding incentivization of innovation, in the premise of BGD, also fosters stimulation of green innovation. Therefore, we hypothesize our second hypothesis as follows:

Hypothesis (H2): There is a positive connection between the corporate quality of CSRD and that of GI.

### Moderating Role of CSRD in the Relationship Between BGD and Corporate GIP

As stated earlier, the studies concerning BGD and green constructs have mixed results whereby different external and internal factors are being identified in different studies for these differing results. Galbreath (2011) has inferred that amid the stereotyping of women as an inefficient entity for the bottom line, the masculine discourse prevails on the boards, and the sway of women is undermined in due process. Cumming and Leung (2021) have inferred different organizational fields of institutional theory, which entices normative, coercive, and mimetic pressures on the environmental sustainability initiatives and their dependence on BGD.

Triana et al. (2014) have termed BGD as a double-edged sword that can either improve or hinder the strategic development of a firm’s green innovation, depending upon the sway and alignment of women on boards. Kochan et al. (2003) have shown that mere number-based BGD may not suffice to improve a firm’s environmental performance, but to reap the fruits of BGD, a holistic view of the BGD needs to be considered in terms of the internal and external characteristics of a firm. Post and Byron (2015) in their meta-analysis relying on the upper echelons theory found that the relationship between gender diversity and firm performance varies by firms’ normative and sociocultural contexts, whereby the relationship is positive in the context of greater gender parity and negative in the context of low gender parity.

As CSRD has been considered an effective tool of legitimization for firms (Muñoz-Torres et al., 2013), its quality will potentially cater to the shocks which shape the differing and mixed results of the BGD–GI relationship. Ayers (2014) has ascertained that disseminating quality disclosures to the stakeholders institutes social resilience within the firm, which leverages it with the perceived organizational legitimacy and shields it from market volatility. In such a case, the firm may be in a better position to withstand the deprived performance ensuing events beyond the firm’s control like exogenous shocks and non-market factors. The quality of CSRD had been considered as an outcome variable in its earlier phase (Boin and Van Eeten, 2013); however, in the extant literature, it has been considered as a process variable which contributes to the dynamic capabilities of a firm to cope with adverse events proactively (Wegener et al., 2019).

In the perspective of the role congruence theory, Amore et al. (2014) have reported the influence of the organizational environment on women’s performance and have fostered that, in order to utilize women’s full potential, one has to tackle the hampering factors which encumber women to attain it. A firm with better CSRD quality fosters that it values the social and communal responsibility at par, and therefore, the communal behavior and gender-specific social performance of women would not be misperceived. It potentially excludes the role incongruity for women on boards and hence leverages a better enabling environment for BGD to contribute to the GI.

Given the constraining factors in the relationship between BGD and GI, quality CSRD has the potential to compensate for them in the purview of role congruence and social resilience theories and instill an enabling context for the positive role of female directors in the GI.

Hypothesis (H3): CSRD positively moderates the relationship between BGD and GI.

## Data, Measurement, and Research Methodology

### Sample and Data

We collected data from A-share Chinese companies listed on the Shanghai and Shenzhen stock exchanges spanning between 2010 and 2019, except for financial institutions. All of the data were acquired from the Chinese Stock Exchange and the Accounting Research Database (CSMAR), which is the major source of information for Chinese-listed companies. CSR information was obtained manually. We excluded companies and years for which information on a particular dataset was unreachable. Hence, we acquired 3,736 firm-year observations after removing the missing observations.

### Green Innovation Performance

Survey questionnaire has been traditionally used for the measurement of green innovation and its performance; however, they have inbuilt self-reporting bias and endogeneity issues (Song et al., 2019). In this study, GIP is used as a dependent variable. The keywords used for evaluating the patents are (1) Green, (2) Environmental, (3) Low carbon, (4) Sustainable, (5) Ecology, (6) Energy-saving, (7) Saving, (8) Clean, (9) Recycling, and (10) Environmental protection (Li et al., 2018; Hong et al., 2020). In this research, the quantity of green patents denotes GIP.

### Board Gender Diversity

We examined board diversity on gender using Blau’s index (Blau, 1977). Gender diversity is measured by the absolute value of the difference between 0.5 and the proportion of female directors (gender diversity) (gender diversity = 1 – (pct women workers)^2^ – (pct men workers)^2^). The resulting variable ranges from 0 to 0.5, with a higher value indicating greater gender diversity. As a robustness check, we replaced Blau’s index with the percent of female directors on board (Naveed et al., 2021). The percent of women on board and Blau’s index are highly correlated (cor = 0.8), and the resulting models are substantively similar.

### Quality of Corporate Social Responsibility Disclosure

Corporate social responsibility disclosure and its strategies have been discussed in past research (Tan et al., 2016; Hu et al., 2019; Rauf et al., 2021b). CSRD is typically explained in terms of a firm’s operating method in acquiring information and compliance with the standard guidelines. This study selects the scenarios of corporate responsibility information from 11 features listed in the “Important information from listed corporations’ CSRD reports” in the CSMAR to quantify the quality of firms’ social responsibility information. The 11 factors can be classified as follows: (1) how the disclosure refers to the Global Reporting Program’s “Sustainability reporting rules”; (2) if this report reveals the relief of shareholders’ needs and privileges; (3) whether this report discloses the security of debtors’ legitimate interests; (4) if it discloses the relief of workers’ rights and dignity; (5) if it discloses the prevention of providers’ needs and interests; (6) if it discloses the protection of clients’ and buyers’ rights and privileges; (7) if it discloses sustainable environmental growth; (8) whether or not something is disclosed regarding the community connections and social welfare commitments; (9) whether the development of a social responsibility framework and anticipated results has been reported or not; (10) if it discloses the components of security manufacturing, and lastly (11) if it discloses the deficiencies of the firm in terms of reporting. Social responsibility disclosure information in the 11 factors has been allocated with a value of 0 for no and a value of 1 for yes. As an outcome, the range of CSR is [0, 11]. Following that, the value of QCSRD is determined by dividing the scores of dissimilar firms by a total of 11 points (Rauf et al., 2021a).

#### Control Variables

The control variables incorporated in this study are obtained from the extant literature as depicted in the study by Hu et al. (2020). The variables are given as follows: (1) a dummy variable for the state ownership status of the firm has been included in the study, keeping in view the special influence of state ownership in the settings of China (Li et al., 2020); (2) board size (BSIZE) is the natural log of the number of directors present on a particular board of directors’ platform (Khalid et al., 2022); (3) board independence (BINDP) is the number of independent directors on the board (Khalid et al., 2022); (4) chief executive officer duality (CEOD) is based on the scenario; if the CEO is also a company’s board chair, it may help establish reliable and indisputable governance, encouraging the CEO’s concentration of power (Fan et al., 2007); as a result, CEO duality may have an impact on GIP and CSRD. A dummy variable for CEO duality is utilized, with 1 representing the CEO as a board chair and 0 as otherwise; (5) firm size (FS) is taken as an indicator of financial performance and credibility and measured using the net income (asset) and employee number (Gavana et al., 2017); (6) the book-to-mark ratio (BMR) concerns the ratio of book value over the market value of the shareholder’s capital (Voinea et al., 2022); (7) capital intensity (CAP) is the ratio of total assets to the operating revenue (Lee, 2010); (8) mandatory CSRD (MAND) represents the presence of mandatory regulation regarding CSRD (Chen et al., 2018); (9) return on assets (ROA), ratio of net income to average total assets (Long, 2018), and finally (10) exports of a company (EXPORTS) is a dummy variable representing whether a firm has exports in a particular year (Galbreath, 2019); and (11) a year dummy and an industry dummy were incorporated (Kim et al., 2019). All of these terms are frequently used in studies of Chinese companies.

#### Empirical Model

We developed the least square dummy variable (LSDV) regression model to test our hypothesis and then used two-stage least square (2SLS) and generalized method of moments (GMM) to investigate further. To reduce endogenous problems, we used straggling explanatory variables to develop empirical models.

The relationship between BGD and corporate GIP is examined using Model 1:


(1) $$G\,I\,P_{\left(i,t\right)}=a+\beta_{1}\,B\,G\,D_{\left(i,t\right)}+\sum_{i=1}^{N}\beta_{n}\,c\,o\,n\,t\,r\,o\,l\,s_{\left(i,t\right)}+\varepsilon_{\left(i,t\right)}$$

Model (2) is used to examine the impact of CSRD on corporate green innovation:


(2) $$G\,I\,P_{\left(i,t\right)}=a+\beta_{2}\,Q\,C\,S\,R\,D_{\left(i,t\right)}+\sum_{i=1}^{N}\beta_{n}\,c\,o\,n\,t\,r\,o\,l\,s_{\left(i,t\right)}+\varepsilon_{\left(i,t\right)}$$

Model (3) is used to examine the impact of CSRD on the relationship between BGD and corporate GIP:


(3) $$G\,I\,P_{\left(i,t\right)}=a+\beta_{3}\,B\,G\,D_{\left(i,t\right)}+\beta_{4}\,Q\,C\,S\,R\,D_{\left(i,t\right)}+\beta_{5}\,B\,G\,D_{\left(i,t\right)}$$

$$\times Q\,C\,S\,R\,D_{\left(i,t\right)}+\sum_{i=1}^{N}\beta_{n}\,c\,o\,n\,t\,r\,o\,l\,s_{\left(i,t\right)}+\varepsilon_{\left(i,t\right)}$$

where GIP refers to green innovation performance; BGD depicts the gender diversity of boards; QCSRD indicates a firm’s quality of corporate social responsibility disclosure; BGD × QCSRD shows the interaction between BGD and QCSRD; i and t denote firm and year, respectively; β denotes the presumed parameter; and ε_(*i*,*t*)_indicates the error term. Controls refer to firm-level control variables.

## Results and Discussion

### Descriptive Statistics

The descriptive statistics of the critical variables are presented in Table 1. GIP has an average value of 10.349. BGD and QCSRD have mean values of 0.207, and 5.928, respectively. In addition, QCSRD is significantly and positively correlated with the GIP. Hence, it is evident that QCSRD is a moderator in the contexts of GIP and BGD. Table 2 shows the correlation among all explanatory variables, including control variables.

**TABLE 1:** Descriptive statistics.

Variables	Mean	SD	Min	Max
GIP	7.082	14.945	0.000	97.000
BGD_BI	0.221	0.159	0.000	0.500
BGD_F	5.39	2.999	0.000	10.000
QCSRD	0.150	0.129	0.000	0.667
BSIZE	2.235	0. 249	1.609	2.890
ROA	0.051	0.040	0.000	0.198
BINDP	3.631	1.094	2.000	13.000
SOE	0.856	0.3551	0.000	1.000
CEOD	0.272	0.445	0.000	1.000
FS	22.742	1.585	19.826	27.386
CAP	2.770	4.862	0.397	36.658
BMR	0.640	0.241	0.056	1.321
MAND	0.269	0.444	0.000	1.000
EXPORTS	0.710	0.54	0.000	1.000

**TABLE 2:** Correlation analysis.

Variables	(1)	(2)	(3)	(4)	(5)	(6)	(7)	(8)	(9)	(10)	(11)	(12)	(13)	(14)
GIP	1													
BGD_BI	0.024	1												
BGD_F	0.037	0.962*	1											
QCSRD	0.087*	−0.032	−0.027	1										
BSIZE	0.101*	−0.077*	−0.109*	0.122*	1									
BINDP	0.082*	−0.038	−0.061*	0.123*	0.760*	1								
SOE	−0.038	0.079*	0.082*	−0.016	−0.170*	−0.136*	1							
CEOD	−0.005	0.116*	0.131*	−0.060*	−0.193*	−0.106*	0.090*	1						
CAP	0.074*	0.026	0.006	0.064*	0.279*	0.287*	−0.019	−0.050*	1					
FSIZE	0.326*	−0.163*	−0.177*	0.378*	0.357*	0.348*	−0.120*	−0.207*	0.374*	1				
BMR	0.132*	−0.099*	−0.108*	0.179*	0.227*	0.186*	−0.113*	−0.159*	0.195*	0.566*	1			
MAND	0.243*	−0.175*	−0.175*	0.401*	0.229*	0.235*	−0.087*	−0.124*	0.196*	0.630*	0.251*	1		
ROA	−0.012	0.077*	0.084*	−0.015	−0.083*	−0.072*	0.086*	0.100*	−0.202*	−0.151*	−0.380*	−0.034	1	
EXPORTS	0.025	−0.005	−0.011	0.011	−0.028	−0.006	−0.031	0.069*	−0.088*	−0.070*	−0.004	−0.04	−0.002	1

***p < 0.01.

**p < 0.05.

*p < 0.1.

### Correlation Matrix

The correlation coefficients of the primary variable’s results are presented in Table 2. The results show that the consistency of GIP and BGD is at a 1% level, and the consistency of QCSRD is at a 5% level. Hence, it can be understood that green innovation and BGD have a positive and significant association with moderating QCSRD, which also shows consistency at the 1% stage within univariate influencing factors. All correlation analyses are below 0.70, suggesting that the maximum correlation among all variables does not increase by 0.50. As a consequence, no multicollinearity problem can have a severe influence on our findings. The correlation coefficient of variance inflation factors (VIFs) is 0.485, indicating no collinearity between variables.

### OLS Regression Results

Table 3 presents least square dummy variable (LSDV) regression results of Equations (1–3). Hypothesis (1) suggests that the board gender diversity has a direct relationship with the green innovation performance of the firm. Model (1), depicting the first hypothesis (H1), shows that BGD is significantly and positively associated with a firm’s GIP (β = 3.4184, *p* < 0.05), which is also in line with the research of Bajic and Yurtoglu (2018). Model 1 was re-estimated through the second measure of BGD in terms of the percentage of women on board, and the results remain positive and significant, depicting the robustness of the study (β = 2.9940, *p* < 0.1).

**TABLE 3:** Main regression results based on LSDV.

Variables	Blau’s index			% of Females	
		
	(1)	(2)	(3)	(4)	(5)
					
	GIP	GIP	GIP	GIP	GIP
BGD (BI)	3.4184**		2.6332		
	(1.3651)		(2.3820)		
QCSRD		0.3555***	0.6361***		0.5055***
		(0.0723)	(0.1264)		(0.1116)
QCSRD*BGD (BI)			1.1914***		
			(0.3939)		
BGD (pcnt)				2.9940*	1.5837
				(1.5823)	(2.8529)
QCSRD*BGD (pcnt)					0.9232*
					(0.4718)
BSIZE	2.8298*	2.8224*	2.8665*	2.8423*	2.9311*
	(1.6311)	(1.6414)	(1.6326)	(1.6317)	(1.6343)
BINDP	−0.8553***	−0.8253**	−0.8510***	−0.8475***	−0.8498***
	(0.3221)	(0.3235)	(0.3221)	(0.3221)	(0.3225)
SOE	−0.1737	−0.0563	−0.0887	−0.1539	−0.0784
	(0.7234)	(0.7215)	(0.7217)	(0.7237)	(0.7219)
CEOD	2.0846***	2.1873***	2.1155***	2.0998***	2.1253***
	(0.5817)	(0.5774)	(0.5804)	(0.5835)	(0.5826)
CAP	−0.1701*	−0.1783*	−0.1951*	−0.1659*	−0.1880*
	(0.0991)	(0.1003)	(0.0998)	(0.0992)	(0.1001)
FSIZE	3.6377***	3.7045***	3.7579***	3.6251***	3.7477***
	(0.3480)	(0.3522)	(0.3529)	(0.3477)	(0.3531)
BMR	−4.4900***	−4.5143***	−4.4671***	−4.4874***	−4.5061***
	(1.0817)	(1.0804)	(1.0798)	(1.0818)	(1.0804)
MAND	1.7106**	2.3700***	2.6786***	1.6652**	2.5618***
	(0.7331)	(0.7513)	(0.7601)	(0.7307)	(0.7560)
ROA	−0.2012	0.7363	−0.7454	0.0596	−0.2745
	(5.3625)	(5.3325)	(5.3590)	(5.3620)	(5.3569)
EXPORTS	1.4842***	1.5866***	1.5744***	1.4888***	1.5915***
	(0.5111)	(0.5108)	(0.5103)	(0.5114)	(0.5109)
Constant	1.5355***	1.0883***	4.9302***	3.1109***	3.5392***
	(2.2983)	(2.2699)	(0.7032)	(0.8608)	(0.8345)
Industry	Included	Included	Included	Included	Included
Year	Included	Included	Included	Included	Included
Observations	3,736	3,736	3,736	3,736	3,736
R-squared	0.1232	0.1259	0.1287	0.1226	0.1272

*Robust standard errors in parentheses.*

***p < 0.01.

**p < 0.05.

*p < 0.1.

Hypothesis (2) suggests that the quality of corporate social responsibility disclosure has a direct relationship with green innovation performance of a firm. Model (2), depicting the second hypothesis (H2), shows that QCSRD positively impacts GIP (β = 0.3555, *p* < 0.01).

Hypothesis (3) suggests that the quality of corporate social responsibility disclosure has a moderating effect on the direct relationship between board gender diversity and green innovation performance of a firm. Following Aiken and West (1991), the moderation term was inculcated in the last model after the investigation of the direct effects. Model 3’s results depict that the coefficient for the interaction term (BGD x CSRD) is positive and significant (β = 1.1914, *p* < 0.01), proving hypothesis 3, which is in line with the research of J. Xu et al. (2020). Model 3 was re-evaluated on the second measure of BGD in terms of the percentage of female directors on board, and the results depict the robustness of the study as the coefficient remains significant and positive (β = 0.9232, *p* < 0.1).

Following Becker’s (2005) research, the control variable, namely absorptive capacity, which has no significant effect on the GIP, was purged from the original model in order to optimize the model for better fit and efficiency.

### Endogeneity Check

To check for the endogeneity problem, we employed two alternative models: a one-year lag measure of BGD and QCRD 2SLS regression as instrumental variables, which shows that the results are robust (Table 4), and a generalized method of moments for the endogeneity check, the results of which are also shown in Table 4.

**TABLE 4:** Regression results based on GMM and 2SLS techniques.

	GMM			2SLS		
		
Variables	(1)	(2)	(3)	(4)	(5)	(6)
						
	GIP	GIP	GIP	GIP	GIP	GIP
BI	5.2189*		3.3482	5.2189*		2.1929
	(2.7002)		(5.0167)	(2.7002)		(5.0279)
QCSRD11		0.9688***	0.7734***		0.9688***	0.7922***
		(0.1713)	(0.2355)		(0.1713)	(0.2357)
QCSRD*BI			1.4656*			1.2766*
			(0.7606)			(0.7635)
BSIZE	4.7452*	4.6494*	5.3780**	4.7452*	4.6493*	4.6508*
	(2.5435)	(2.5790)	(2.5257)	(2.5435)	(2.5790)	(2.5466)
BINDP	−1.3986***	−1.3488***	−1.4985***	−1.3986***	−1.3488***	−1.3729***
	(0.4867)	(0.4930)	(0.4830)	(0.4867)	(0.4930)	(0.4875)
SOE	−0.5089	−0.2657	−0.3940	−0.5090	−0.2657	−0.4155
	(1.1505)	(1.1532)	(1.1602)	(1.1505)	(1.1532)	(1.1608)
CEOD	2.7966***	3.1452***	2.7787***	2.7966***	3.1452***	2.8827***
	(0.9483)	(0.9516)	(0.9553)	(0.9483)	(0.9516)	(0.9543)
CAP	−0.1675	−0.1806	−0.1718	−0.1675	−0.1806	−0.1760
	(0.1456)	(0.1473)	(0.1471)	(0.1456)	(0.1473)	(0.1471)
FSIZE	5.0261***	5.1683***	4.9983***	5.0261***	5.1683***	5.1055***
	(0.5489)	(0.5546)	(0.5609)	(0.5489)	(0.5546)	(0.5616)
BMR	−6.7781***	−5.9825***	−5.6199***	−6.7781***	−5.9825***	−5.7262***
	(1.8436)	(1.8260)	(1.9715)	(1.8436)	(1.8260)	(1.9715)
MAND	0.9679	2.5438**	1.9877*	0.9680	2.5438**	1.9407*
	(1.0402)	(1.0403)	(1.0605)	(1.0402)	(1.0403)	(1.0616)
ROA	−1.4499	2.2604	6.5407	−1.4499	2.2605	5.7890
	(9.9918)	(9.8951)	(13.5884)	(9.9918)	(9.8951)	(13.6074)
EXPORTS	2.5162***	2.7141***	2.4312***	2.5162***	2.7140***	2.6001***
	(0.8490)	(0.8473)	(0.8437)	(0.8490)	(0.8472)	(0.8463)
Constant	4.2129***	2.6357***	3.1095***	4.2635***	2.7633***	3.7051***
	(0.3386)	(0.4313)	(0.5179)	(0.36293)	(3.1680)	(0.75719)
Industry	Included	Included	Included	Included	Included	Included
Year	Included	Included	Included	Included	Included	Included
Observations	1,975	1,975	1,975	1,975	1,975	1,975
R-squared	0.1456	0.1439	0.1507	0.1456	0.1439	0.1510

*Robust standard errors in parentheses.*

***p < 0.01.

**p < 0.05.

*p < 0.1.

## Discussion

This study coalesces the social resilience and role congruence theories to investigate the upshot of quality of corporate social responsibility disclosure on the relationship between board gender diversity and corporate green innovation performance. We explored a broad data set to search the existing literature by (1) predicating the notion that the firm-level indicator of QCSRD has an encompassing manifestation of the social resilience of the firm to cater to the constraining impacts in the BGD–GIP relationship and by (2) promulgating a role congruence-leveraged capability-based logic that explicates the varied impacts of BGD on the corporate GIP. Consequently, this study is derived from the extant literature to propose a firm-level indicator for the role congruence of board members in terms of their gender, fostering the social resilience of a firm.

Pertaining to our first hypothesis, our findings are supportive of the notion of a direct relationship between BGD and corporate GIP for the provided sample. It confirms the positive role of female members on board in the promotion of “Going greener” agenda on board amid their communal acumen, perceptive skills, and participative leadership style.

Pertaining to our second hypothesis, our findings are also supportive of the significant relationship between the quality of CSRD and corporate GIP for the provided sample. It confirms the positive association of the quality of CSRD with the enhanced consideration of social responsibility, compliance with the global standards, and mitigation of the principal–agent dilemma on the part of the firms.

Pertaining to our third hypothesis, our findings support the moderating role of the quality of CSRD in the relationship between BGD and GIP of firms. It confirms the positive interaction of the quality of CSRD with the enhanced capacity of firms to absorb exogenous shocks beyond firms’ control and leverage a better enabling environment for women on board to contribute to the green innovation performance of firms.

### Theoretical Contributions

First, the study expands the existing theoretical purview by explicating firms’ existence in the perspective of the prerogatives of the society. For instance, existing research theorizes the BGD–GIP relationship in terms of internal factors, contextual factors, and institutional settings in which the firms operate. Both the legitimacy and stakeholder theories describe the relationship as a response to the enticed pressures from these factors through a resource-based view. In extension to this view, a remarkable point of the current study is that it asserts the role congruence of women on board in terms of a conducive environment for women’s gender-specific performance (Amore et al., 2014). Role congruence complements the resource base for the optimized capability of the firm, on the one hand, and tackles the hampering factors restraining women to deliver at their full potential, on the other hand, so as to address the demands regarding the “Going greener” initiatives and innovations (Triana et al., 2014; Vial et al., 2016).

A firm with better CSRD is an indicator that the firm values the societal prerogative of a firm, and therefore, the communal conduct and gender-specific performance of women would not be misconstrued. It excludes the role incongruity for women on boards while leveraging a better enabling environment for BGD to contribute to the GI. Thus, the first input to the theory regarding the BGD–GIP relationship is that accounting for internal, contextual, and institutional pressures does not suffice for the complete description of the phenomenon. More indicatively, the better quality of CSRD encompasses the amelioration of an enabling environment for the relationship and mitigating the constraining factors whatsoever.

Second, while the quality of CSRD can indicate the role congruence of women on board for their full potential delivery, there may be unexpected exogenous shocks that hamper the green innovation performance and alter the BGD–GIP relationship. The current study has built the premise of organizational resilience on the propositions of Ayers (2014) and Wegener et al. (2019). Ayers (2014) has argued the social resilience of an organization in terms of the quality of CSRD for market volatility, while Wegener et al. (2019) have considered it as an outcome of the dynamic capabilities of a firm. The findings of the current study profess the moderating role of the quality of CSRD in terms of organizational resilience to cope with these shocks. Quality CSRD, a dynamic capability and a tool of legitimization of a firm, would leverage the organizational resilience and catre to the shocks which shape the differing and mixed results of the BGD–GI relationship (Muñoz-Torres et al., 2013).

### Practical Contributions

Managers attempting to improve green innovation performance may consider BGD in the purview of the quality of CSRD. According to this study, higher levels of CSR information may boost the impact of BGD on corporate GIP performance, which has important practical implications for practitioners looking for corporate GIP. The role of CSRD may be considered as an indicator of the conduciveness for the stand-alone version of GIP and also the BGD-leveraged GIP. The outcomes of this research enable the firms to engage in CSR strategies that deliver green signals to internal and external stakeholders, resulting in green outcomes. Therefore, business executives need to focus on the interplay between QCSRD, BGD, and GIP so as to provide the underpinning for the amelioration of green innovation performance of a firm. This study also underscores the need for highlighting the quality performance of a firm in terms of CSRD to garner legitimacy for itself and also disseminate the signal of green compliance to the relevant stakeholders.

At the policy level, the government also needs to support the firms in order to encourage them to improve their quality in terms of CSRD and disclose it vehemently. This will ease their capital financing, and they will be able to attract green finance. To assure the authority of CSR rating conclusions, the government should establish a supervisory mechanism and share CSR information with environmental protection departments while mandating it as a criterion for qualification for the corporate GIP subsidy.

## Conclusion

Corporate social responsibility disclosure has progressively qualified itself as a requirement for all businesses. This empirical study focuses on board gender diversity and quality of CSRD while considering corporate green innovation performance in China. With a systematic examination of CSR reports under the social resilience theory, this is the first exploratory study of this sort in the premise of role congruence theory’s underpinning of BGD. The study is based on panel data of 12,464 Chinese firm-year observations from 2014 to 2020. Corporate GIP has been demonstrated to be boosted by BGD in enterprises. Companies having a higher QCSRD are more likely to engage in corporate GIP, and it has a prominent moderating effect on the relationship between BGD and GIP. Practical implications of this research assist managers in making decisions, resulting in improved organizational reputation and innovation performance under the “Go green” perspective. The findings of this study contribute to the body of CSR research by examining the impact of QCSRD on corporate GIP and the relationship between BGD and GIP.

Future research could be guided by the limitations of this study. To begin with, our findings confirm the existence of a favorable link between corporate GIP and CSRD, and we acknowledge that there is still a niche for future research to expand on our findings and devise a framework for the interplay of CSRD with the relevant antecedents. Second, to measure the variable of BGD, we utilized Blau’s index and the percentage of women on board; however, there are other perspectives of gender diversity in terms of different other indices which can be explored.

Third, corporations intervene in community collaboration, particularly within CSR implementation, since there are issues with functional limitations and the inability to conduct CSR. The limitations to the quality of CSR and its relevance with the green-oriented innovation may enable the society to participate in the implementation of CSR through the production of various environment-friendly products, thereby helping to reduce social and environmental problems. The engagement in social participation and firm’s corporate GIP are more likely to increase environmental sustainability and social welfare. The findings of the analysis can be utilized as a foundation for future research to test and construct more intricate models while incorporating the antecedents of the quality of CSRD.

Lastly, we have concentrated on Chinese firms for the exploration of our hypothesis, so the research results may not apply to other countries. The comparative analysis of different institutional settings may explore the phenomenon with further intricacies. Therefore, future research may focus on comparative studies within developing countries and between developed and developing countries.

## Data Availability Statement

The data analyzed in this study is subject to the following licenses/restrictions: Restricted by issuing authority. Requests to access these datasets should be directed to http://cndata1.csmar.com.

## Author Contributions

KN and CV conceived the idea. KN wrote the introduction, literature review, and empirical outcome sections. CV and NR helped with the methodology, analysis, and discussion sections. KN accomplished the write-up. CV carried out proofreading. All authors read and approved the final manuscript.

## Conflict of Interest

The authors declare that the research was conducted in the absence of any commercial or financial relationships that could be construed as a potential conflict of interest.

## Publisher’s Note

All claims expressed in this article are solely those of the authors and do not necessarily represent those of their affiliated organizations, or those of the publisher, the editors and the reviewers. Any product that may be evaluated in this article, or claim that may be made by its manufacturer, is not guaranteed or endorsed by the publisher.

## References

[B1] Adams R. B. Ferreira D. (2009). Women in the boardroom and their impact on governance and performance. *J. Financ. Econ.* 94 291–309. 10.1016/j.jfineco.2008.10.007 11184009

[B2] Aghion P. Van Reenen J. Zingales L. (2013). Innovation and institutional ownership. *Am. Econ. Rev.* 103 277–304. 10.1257/aer.103.1.277

[B3] Aiken L. S. West S. G. (1991). *Multiple Regression: Testing and Interpreting Interactions.* Thousand Oaks, CA: Sage Publications, Inc.

[B4] Amore M. D. Garofalo O. Minichilli A. (2014). Gender interactions within the family firm. *Manag. Sci.* 60 1083–1097. 10.1287/mnsc.2013.1824 19642375

[B5] Ayers J. (2014). *The Importance of Social Resilience in Organizations.* Available online at: http://drcaroladams.net/the-importance-of-social-resilience-in-organisations/ (accessed September 10, 2021).

[B6] Bajic S. Yurtoglu B. (2018). Which aspects of CSR predict firm market value? *J. Capital Markets Stud.* 2 50–69. 10.1108/JCMS-10-2017-0002

[B7] Bannò M. Filippi E. Trento S. (2021). Women in top echelon positions and their effects on sustainability: a review, synthesis and future research agenda. *J. Manag. Governance* [Epub ahead of print]. 10.1007/s10997-021-09604-7

[B8] Bansal P. Clelland I. (2004). Talking trash: legitimacy, impression management, and unsystematic risk in the context of the natural environment. *Acad. Manag. J.* 47 93–103. 10.5465/20159562 20159562

[B9] Bartlett D. Trifilova A. (2010). Green technology and eco-innovation: seven case-studies from a Russian manufacturing context. *J. Manuf. Technol. Manag.* 21 910–929. 10.1108/17410381011086757

[B10] Becker T. E. (2005). Potential problems in the statistical control of variables in organizational research: a qualitative analysis with recommendations. *Organ. Res. Methods* 8, 274–289. 10.1177/1094428105278021

[B11] Berrone P. Fosfuri A. Gelabert L. Gomez-Mejia L. R. (2013). Necessity as the mother of ‘green’inventions: institutional pressures and environmental innovations. *Strateg. Manag. J.* 34 891–909. 10.1002/smj.2041

[B12] Blau P. M. (1977). *Inequality and Heterogeneity: A Primitive Theory of Social Structure.* New York, NY: Free Press, 677–683.

[B13] Boin A. Van Eeten M. J. (2013). The resilient organization. *Public Manag. Rev.* 15 429–445. 10.1080/14719037.2013.769856

[B14] Bradley S. W. Klein P. (2016). Institutions, economic freedom, and entrepreneurship: the contribution of management scholarship. *Acad. Manag. Perspect.* 30 211–221. 10.5465/amp.2013.0137

[B15] Castellacci F. Lie C. M. (2017). A taxonomy of green innovators: empirical evidence from South Korea. *J. Clean. Product.* 143 1036–1047. 10.1016/j.jclepro.2016.12.016

[B16] Chang V. De Roure D. Wills G. John Walters R. Barry T. (2011). Organisational sustainability modelling for return on investment (ROI): case studies presented by a national health service (NHS) trust UK. *J. Comput. Inform. Technol.* 19 177–192. 10.2498/cit.1001951

[B17] Chen L. Srinidhi B. Tsang A. Yu W. (2016). Audited financial reporting and voluntary disclosure of corporate social responsibility (CSR) reports. *J. Manag. Account. Res.* 28 53–76. 10.2308/jmar-51411

[B18] Chen Y.-C. Hung M. Wang Y. (2018). The effect of mandatory CSR disclosure on firm profitability and social externalities: evidence from China. *J. Account. Econ.* 65 169–190. 10.1016/j.jacceco.2017.11.009

[B19] Chen Y. S. Lai S. B. Wen C. T. (2006). The influence of green innovation performance on corporate advantage in Taiwan. *J. Bus. Ethics* 67 331–339. 10.1007/s10551-006-9025-5

[B20] Chiu S. C. Sharfman M. (2011). Legitimacy, visibility, and the antecedents of corporate social performance: an investigation of the instrumental perspective. *J. Manag.* 37 1558–1585. 10.1177/0149206309347958

[B21] Cho C. H. Patten D. M. (2013). Green accounting: reflections from a CSR and environmental disclosure perspective. *Crit. Perspect. Account.* 24 443–447. 10.1016/j.cpa.2013.04.003

[B22] Chu Z. Wang L. Lai F. (2018). Customer pressure and green innovations at third party logistics providers in China: the moderation effect of organizational culture. *Int. J. Logist. Manag.* 30 57–75. 10.1108/IJLM-11-2017-0294

[B23] Coutu D. L. (2002). How resilience works. *Harv. Bus. Rev.* 80 46–56. 12024758

[B24] Cumming D. Leung T. Y. (2021). Board diversity and corporate innovation: regional demographics and industry context. *Corporate Govern. Int. Rev.* 29 277–296. 10.1111/corg.12365

[B25] Deegan C. (2002). Introduction: the legitimising effect of social and environmental disclosures–a theoretical foundation. *Account. Audit. Account. J.* 15 282–311. 10.1108/09513570210435852

[B26] Dhaliwal D. S. Li O. Z. Tsang A. Yang Y. G. (2011). Voluntary nonfinancial disclosure and the cost of equity capital: the initiation of corporate social responsibility reporting. *Account. Rev.* 86 59–100. 10.2308/accr.00000005

[B27] Dixon-Fowler H. R. Ellstrand A. E. Johnson J. L. (2017). The role of board environmental committees in corporate environmental performance. *J. Bus. Ethics* 140 423–438. 10.1007/s10551-015-2664-7

[B28] Duchek S. (2014). Growth in the face of crisis: the role of organizational resilience capabilities. *Acad. Manag. Proc.* 2014:13487. 10.5465/ambpp.2014.225

[B29] Fan D. K. Lau C.-M. Young M. (2007). Is China’s corporate governance beginning to come of age? The case of CEO turnover. *Pac. Basin Finance J.* 15 105–120. 10.1016/j.pacfin.2006.08.001

[B30] Freeman S. (2007). *Rawls.* London: Routledge. 10.4324/9780203086605

[B31] Frondel M. Horbach J. Rennings K. (2008). What triggers environmental management and innovation? Empirical evidence for Germany. *Ecol. Econ.* 66 153–160. 10.1016/j.ecolecon.2007.08.016

[B32] Galbreath J. (2011). Are there gender-related influences on corporate sustainability? A study of women on boards of directors. *J. Manag. Organ.* 17 17–38. 10.5172/jmo.2011.17.1.17

[B33] Galbreath J. (2019). Drivers of green innovations: the impact of export intensity, women leaders, and absorptive capacity. *J. Bus. Ethics* 158 47–61. 10.1007/s10551-017-3715-z

[B34] García-Sánchez I. M. Suárez-Fernández O. Martínez-Ferrero J. (2019). Female directors and impression management in sustainability reporting. *Int. Bus. Rev.* 28 359–374. 10.1016/j.ibusrev.2018.10.007

[B35] Gavana G. Gottardo P. Moisello A. M. (2017). Earnings management and CSR disclosure. Family vs. non-family firms. *Sustainability* 9:2327. 10.3390/su9122327

[B36] Glass C. Cook A. (2016). Leading at the top: understanding women’s challenges above the glass ceiling. *Leadersh. Q.* 27 51–63. 10.1016/j.leaqua.2015.09.003

[B37] Gray R. Kouhy R. Lavers S. (1995). Corporate social and environmental reporting. *Account. Audit. Account. J.* 8 47–77. 10.1108/09513579510146996

[B38] Hambrick D. C. (2007). Upper echelons theory: an update. *Acad. Manag. Rev.* 32 334–343. 10.5465/amr.2007.24345254

[B39] Hillman A. J. Cannella A. A. Paetzold R. L. (2000). The resource dependence role of corporate directors: strategic adaptation of board composition in response to environmental change. *J. Manag. Stud.* 37 235–256. 10.1111/1467-6486.00179

[B40] Hillman A. J. Dalziel T. (2003). Boards of directors and firm performance: integrating agency and resource dependence perspectives. *Acad. Manag. Rev.* 28 383–396. 10.2307/30040728

[B41] Hong M. Drakeford B. Zhang K. (2020). The impact of mandatory CSR disclosure on green innovation: evidence from China. *Green Finan.* 2 302–322. 10.3934/GF.2020017

[B42] Hu H. Dou B. Wang A. (2019). Corporate Social responsibility information disclosure and corporate fraud—“risk reduction” effect or “window dressing” effect? *Sustainability* 11:1141. 10.3390/su11041141

[B43] Hu W. Du J. Zhang W. (2020). Corporate social responsibility information disclosure and innovation sustainability: evidence from China. *Sustainability* 12:409. 10.3390/su12010409

[B44] Hülsbeck M. Meoli M. Vismara S. (2019). The board value protection function in young, mature and family firms. *Br. J. Manag.* 30 437–458. 10.1111/1467-8551.12322

[B45] Issa A. Fang H. X. (2019). The impact of board gender diversity on corporate social responsibility in the Arab Gulf states. *Gend. Manag. Int. J.* 34 577–605. 10.1108/GM-07-2018-0087

[B46] Kawai N. Strange R. Zucchella A. (2018). Stakeholder pressures, EMS implementation, and green innovation in MNC overseas subsidiaries. *Int. Bus. Rev.* 27 933–946. 10.1016/j.ibusrev.2018.02.004

[B47] Khalid F. Naveed K. Nawaz R. Sun X. Wu Y. Ye C. (2022). Does corporate green investment enhance profitability? An institutional perspective. *Econ. Res. Ekonomska Istraživanja* [Epub ahead of print]. 10.1080/1331677X.2022.2063919

[B48] Kim D. Starks L. T. (2016). Gender diversity on corporate boards: do women contribute unique skills? *Am. Econ. Rev.* 106 267–271. 10.1257/aer.p20161032

[B49] Kim J. Cho K. Park C. K. (2019). Does CSR assurance affect the relationship between CSR performance and financial performance? *Sustainability* 11:5682. 10.3390/su11205682

[B50] Kochan T. Bezrukova K. Ely R. Jackson S. Joshi A. Jehn K. (2003). The effects of diversity on business performance: report of the diversity research network. *Hum. Resour. Manag.* 42 3–21. 10.1002/hrm.10061

[B51] Lee S. (2010). Effects of Capital intensity on firm performance: the US Restaurant industry. *J. Hosp. Financ. Manag.* 18 1–13. 10.1080/10913211.2010.10653882

[B52] Li D. Huang M. Ren S. Chen X. Ning L. (2018). Environmental legitimacy, green innovation, and corporate carbon disclosure: evidence from CDP China 100. *J. Bus. Ethics* 150 1089–1104. 10.1007/s10551-016-3187-6

[B53] Li H. Hang Y. Shah S. G. M. Akram A. Ozturk I. (2020). Demonstrating the impact of cognitive CEO on firms’ performance and CSR activity. *Front. Psychol.* 11:278. 10.3389/fpsyg.2020.00278 32184747 PMC7058782

[B54] Lindblom C. K. (1994). “The implications of organizational legitimacy for corporate social performance and disclosure,” in *Proceedings of the Critical Perspectives on Accounting Conference*, New York, NY.

[B55] Long D. A. (2018). *Analysing the Influence of Return on Assets, Return on Equity and Non-Performing Laons Ratio Toward Gross Domestic Product Growth Rate in China (A Case of Study China’s Commercial Banks 2012-2016).* Doctoral dissertation. West Java: President University.

[B56] Lopatta K. Böttcher K. Lodhia S. K. Tideman S. A. (2020). The relationship between gender diversity and employee representation at the board level and non-financial performance: a cross-country study. *Int. J. Account.* 55:2050001. 10.1142/S1094406020500018

[B57] Miller T. del Carmen Triana M. (2009). Demographic diversity in the boardroom: mediators of the board diversity–firm performance relationship. *J. Manag. Stud.* 46 755–786. 10.1111/j.1467-6486.2009.00839.x

[B58] Milliken F. J. Martins L. L. (1996). Searching for common threads: understanding the multiple effects of diversity in organizational groups. *Acad. Manag. Rev.* 21 402–433. 10.5465/amr.1996.9605060217

[B59] Muñoz-Torres M. J. Fernandez-Izquierdo M. A. Rivera-Lirio J. M. León Soriano R. Escrig-Olmedo E. Ferrero-Ferrero I. (2013). Materiality analysis for CSR reporting in Spanish SMEs. *Int. J. Manag. Knowl. Learn.* 1 231–250.

[B60] Nadeem W. Juntunen M. Shirazi F. Hajli N. (2020). Consumers’ value co-creation in sharing economy: the role of social support, consumers’ ethical perceptions and relationship quality. *Technol. Forecast. Soc. Change* 151:119786. 10.1016/j.techfore.2019.119786

[B61] Naveed K. Voinea C. L. Ali Z. Rauf F. Fratostiteanu C. (2021). Board gender diversity and corporate social performance in different industry groups: evidence from China. *Sustainability* 13:3142. 10.3390/su13063142

[B62] Nguyen T. H. Elmagrhi M. H. Ntim C. G. Wu Y. (2021). Environmental performance, sustainability, governance and financial performance: evidence from heavily polluting industries in China. *Bus. Strategy Environ.* 30 2313–2331. 10.1002/bse.2748

[B63] Nielsen S. Huse M. (2010). The contribution of women on boards of directors: going beyond the surface. *Corp. Gov. Int. Rev.* 18 136–148. 10.1111/j.1467-8683.2010.00784.x

[B64] Orazalin N. Baydauletov M. (2020). Corporate social responsibility strategy and corporate environmental and social performance: the moderating role of board gender diversity. *Corp. Soc. Responsib. Environ. Manag.* 27 1664–1676. 10.1002/csr.1915

[B65] Orazalin N. Mahmood M. (2021). Toward sustainable development: board characteristics, country governance quality, and environmental performance. *Bus. Strategy Environ.* 30 3569–3588. 10.1002/bse.2820

[B66] Pisano G. P. Shih W. C. (2012). *Producing Prosperity: Why America Needs a Manufacturing Renaissance.* Boston, MA: Harvard Business Press.

[B67] Post C. Byron K. (2015). Women on boards and firm financial performance: a meta-analysis. *Acad. Manag. J.* 58 1546–1571. 10.5465/amj.2013.0319 35019162

[B68] Post C. Rahman N. McQuillen C. (2015). From board composition to corporate environmental performance through sustainability-themed alliances. *J. Bus. Ethics* 130 423–435. 10.1007/s10551-014-2231-7

[B69] Post C. Rahman N. Rubow E. (2011). Green governance: boards of directors’ composition and environmental corporate social responsibility. *Bus. Soc.* 50 189–223. 10.1177/0007650310394642

[B70] Ramos A. R. Ferreira J. C. E. Kumar V. Garza-Reyes J. A. Cherrafi A. (2018). A lean and cleaner production benchmarking method for sustainability assessment: a study of manufacturing companies in Brazil. *J. Clean. Product.* 177 218–231. 10.1016/j.jclepro.2017.12.145

[B71] Rauf F. Voinea C. L. Naveed K. Fratostiteanu C. (2021a). CSR disclosure: effects of political ties, executive turnover and shareholder equity. Evidence from China. *Sustainability* 13:3623. 10.3390/su13073623

[B72] Rauf F. Voinea C. L. Roijakkers N. Naveed K. Hashmi H. B. A. Rani T. (2021b). How executive turnover influences the quality of corporate social responsibility disclosure? Moderating role of political embeddedness: evidence from China. *Eurasian Bus. Rev.* [Epub ahead of print]. 10.1007/s40821-021-00187-9

[B73] Rehbein K. Logsdon J. M. Van Buren H. J. (2013). Corporate responses to shareholder activists: considering the dialogue alternative. *J. Bus. Ethics* 112 137–154. 10.1007/s10551-012-1237-2

[B74] Reichstein T. Salter A. (2006). Investigating the sources of process innovation among UK manufacturing firms. *Industr. Corp. Change* 15 653–682. 10.1093/icc/dtl014 30476010

[B75] Robinson J. L. Lipman-Blumen J. (2003). Leadership behavior of male and female managers, 1984–2002. *J. Educ. Bus.* 79 28–33. 10.1080/08832320309599084

[B76] Sheffi Y. Rice J. B. Jr. (2005). A supply chain view of the resilient enterprise. *MIT Sloan Manag. Rev.* 47:41. 34728899

[B77] Song M. Fisher R. Kwoh Y. (2019). Technological challenges of green innovation and sustainable resource management with large scale data. *Technol. Forecast. Soc. Change* 144 361–368. 10.1016/j.techfore.2018.07.055

[B78] Tan A. Benni D. Liani W. (2016). Determinants of corporate social responsibility disclosure and investor reaction. *Int. J. Econ. Financ. Issues* 6 11–17.

[B79] Triana M. D. C. Miller T. L. Trzebiatowski T. M. (2014). The double-edged nature of board gender diversity: diversity, firm performance, and the power of women directors as predictors of strategic change. *Organ. Sci.* 25 609–632. 10.1287/orsc.2013.0842 19642375

[B80] Vial A. C. Napier J. L. Brescoll V. L. (2016). A bed of thorns: female leaders and the self-reinforcing cycle of illegitimacy. *Leadersh. Q.* 27 400–414. 10.1016/j.leaqua.2015.12.004

[B81] Voinea C. L. Rauf F. Naveed K. Fratostiteanu C. (2022). The impact of CEO duality and financial performance on CSR disclosure: empirical evidence from state-owned enterprises in China. *J. Risk Financ. Manag.* 15:37. 10.3390/jrfm15010037

[B82] Wang H. Tong L. Takeuchi R. George G. (2016). *Corporate Social Responsibility: An Overview and New Research Directions: Thematic Issue on Corporate Social Responsibility.* Briarcliff Manor, NY: Academy of Management. 10.5465/amj.2016.5001

[B83] Wegener M. Labelle R. Jerman L. (2019). Unpacking carbon accounting numbers: a study of the commensurability and comparability of corporate greenhouse gas emission disclosures. *J. Clean. Product.* 211 652–664. 10.1016/j.jclepro.2018.11.156

[B84] Xu S. Chen X. Li A. Xia X. (2020). Disclosure for whom? Government involvement, CSR disclosure and firm value. *Emerg. Markets Rev.* 44:100717. 10.1016/j.ememar.2020.100717

[B85] Zahller K. A. Arnold V. Roberts R. W. (2015). Using CSR disclosure quality to develop social resilience to exogenous shocks: a test of investor perceptions. *Behav. Res. Account.* 27 155–177. 10.2308/bria-51118

